# Key Questions on the Long-Term Utility of Methylphenidate in Paediatric Brain Tumour Survivorship: A Retrospective Clinical Case Series

**DOI:** 10.3390/children11020187

**Published:** 2024-02-02

**Authors:** Alexander J. Hagan, Sarah J. Verity

**Affiliations:** 1 Department of Paediatric Health Psychology, Great North Children’s Hospital, Newcastle upon Tyne Hospitals NHS Foundation Trust, Newcastle upon Tyne NE1 4LP, UK; 2 Newcastle University Centre for Cancer, Newcastle upon Tyne NE1 7RU, UK

**Keywords:** brain tumour, cancer, quality of survival, survivorship, methylphenidate, cognition

## Abstract

Methylphenidate has an established role in the management of attention-deficit hyperactivity disorder and attentional deficit secondary to brain injury. Increasingly, methylphenidate is considered for the attentional deficit in paediatric brain tumour survivors. A small number of studies have explored the benefit of methylphenidate in this population; however, studies are of short duration and do not address the impact of medium to long-term use of methylphenidate on intellectual function. We identified six patients who are survivors of a paediatric brain tumour aged 12–18 years with greater than three years of use of methylphenidate for inclusion in a clinical case series. We used this patient cohort to identify key questions to inform a future long-term cohort study. Linear mixed model and reliable change index analyses were performed on the data. Reliable change index analyses showed benefits to working memory (*n* = 3), processing speed (*n* = 2), and full-scale IQ (*n* = 4) performance for some patients. This exploratory case series suggests the potential medium to long-term benefit of methylphenidate in brain tumour survivorship, indicating the need for larger, appropriately powered studies. These patient data, alongside a discussion of learning points from our previously published studies, are used as a conduit for the identification of questions relating to the use of methylphenidate in a paediatric brain tumour.

## 1. Introduction

Psychostimulant drugs have an established role in the symptom management of children and young people with attention-deficit hyperactivity disorder (ADHD) [1,2]. Supporting the upregulation of cortical function, such drugs are increasingly employed for children with an attentional deficit secondary to traumatic brain injury [3]. The multi-phase study of Conklin et al. highlights the potential utility of methylphenidate for acquired brain injury via a paediatric brain tumour and provides a foundational evidence base for its function in the early stages of survivorship [4,5,6]. Their randomised controlled trial shows methylphenidate to effectively mediate treatment-related processing speed and attentional deficits whilst producing minimal unwanted side effects. Alongside measurable cognitive benefits, methylphenidate has been shown to be highly favourable to patients and parents and to produce a marked increase in the quality of survivorship [7,8].

**Figure 1:**
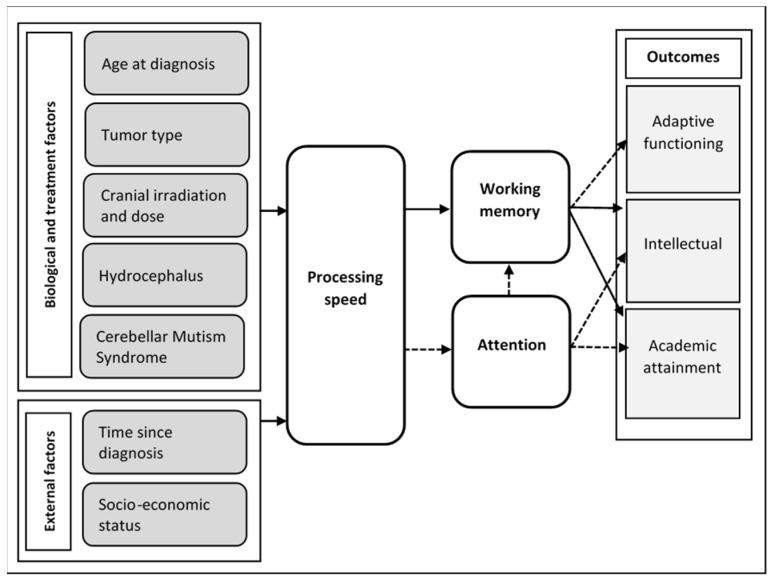
Proposed conceptual model illustrating the role of processing speed and attention in survivorship outcomes following pediatric medulloblastoma. Conceptual model developed by Palmer [11]. Alt text: A model showing the impact of disease and treatment on intellectual outcome and academic attainment. The model shows that processing speed, attention, and working memory are implicated in the relationship between treatment and eventual intellectual and academic outcomes. Solid lines: Associations made from the present literature. Dotted lines: Areas identified for future research. This model suggests that processing speed and attention directly impact working memory ability and indirectly (via memory) impact intellectual outcome.

Whilst the beneficial effect of methylphenidate on attention is well established in this population, the effect on downstream functions dependent on the sustained maintenance of normal attentional ability has not been confirmed. It is highly likely that intact attentional function plays a role in the later development of intellectual ability in childhood via the contribution of attention to working memory function [9,10]. This is consistent with the premise of Palmer, whose proposed conceptual model submits that the intellectual and academic deficit in survivors of childhood medulloblastoma is substantially attributable to impaired processing speed and attention via working memory [11] (see Figure 1). Such models suggest that effective management of attentional deficit may result in a later reduction in the intellectual deficit in survivors of a paediatric brain tumour.

While Palmer offers a strong theoretical justification for the utility of methylphenidate in mediating the effects of tumour and treatment on later intellectual development in childhood, this theory has not been proven clinically. Evidence of the effect of longer-term use of methylphenidate in the brain tumour population is limited. Three published studies report on the effects of this treatment in cohorts at 12 months of use, two of these sourcing the same participant pool [12,13,14]. Studies of participants at 12 months showed improvement in attention as measured by the Conners’ Continuous Performance Task [12] and on selective attention using the Test of Everyday Attention for Children [14]. No improvement was found in measures of intellectual ability as measured via IQ score, and no improvement was found in academic attainment in the one study that measured this [13].

Despite the sensible theoretical rationale for the use of methylphenidate in ameliorating later effects, studies of the impact of methylphenidate in children with ADHD do not offer compelling evidence of the benefit of methylphenidate in either long-term intellectual or academic outcomes. While remaining the recommended first-line treatment for severe ADHD in childhood [15], studies evaluating the effect of long-term use of methylphenidate on intellectual function do not show strong positive benefits [16]. Nor is there unequivocal high-quality evidence for the role of long-term methylphenidate in supporting academic attainment [17]. In fact, higher quality reviews were more likely to find no or little benefit of methylphenidate on long-term academic outcomes, showing methylphenidate to have a greater effect on the level of productivity compared to outcome [18]. Evidence for the benefit of long-term use of methylphenidate in managing ADHD is limited to periods of up to two years [1,19]; however, studies of prescribing trends in ADHD show that over 60% of children continue to use methylphenidate for longer than this period [20,21]. Discontinuation studies considering the efficacy and effect size of the continued use of methylphenidate in the ADHD population suggest that the beneficial effect of methylphenidate is reduced over the longer term [22,23,24,25].

In the context of recent studies highlighting the potential overestimation of the benefit of methylphenidate and underestimation of ‘non-serious’ side effects in ADHD, an evidence-based approach to the long-term use of this drug in our own clinical population is clearly indicated [26]. While the study of Man et al. shows the relative safety of methylphenidate used for up to two years in patients with ADHD, there is a dearth of information weighing the relative benefit of long-term methylphenidate against low-level side effects in the brain tumour population [19].

The current case series describes the trajectory of intellectual development in six survivors of a paediatric brain tumour over a minimum of three years of use of methylphenidate. These patient data, alongside a discussion of learning points from our previously published studies, were used as a conduit for identifying salient questions relating to the use of methylphenidate in a paediatric brain tumour [7,8,14]. These questions will inform our analyses of a study in progress with a larger cohort.

## 2. Materials and Methods

### 2.1. Design

The current study employed a case series design. While the design has obvious methodological limitations in testing the causal inference between treatment and outcome, it is highly useful in hypothesis generation and observing trends to inform future follow-up studies. The JBI Critical Appraisal Checklist for Case Series tool was used to promote methodological validity [27].

### 2.2. Clinical Sample

Participant data in the current retrospective clinical case series were derived from a previous service evaluation study assessing the utility of methylphenidate in managing the attentional deficit in survivors of a paediatric brain tumour [14]. Participant selection for suitability of methylphenidate treatment in this study and associated demographic details have been described previously [7,8,14]. In brief, eligible patients for the service evaluation were aged between 5.0 and 15.5 years at recruitment; had a General Ability Index ≥ 50; had a brain tumour; had completed initial cancer treatment at least 12 months prior to baseline assessment; and had hydrocephalus at diagnosis and/or received cranial radiotherapy. Exclusion criteria included all medical and psychological contraindications to methylphenidate hydrochloride in children, children with a pre-tumour diagnosis of attention-deficit hyperactivity disorder (ADHD), and children whose English language proficiency did not allow them to access neurocognitive measures. These criteria are presented as Appendix A. A total of 87 children were assessed for eligibility between April 2017 and February 2020, of which 30 were eligible for methylphenidate treatment.

A potential case series cohort was created from this sample of 30 patients. Alongside meeting the eligibility criteria for the previous service evaluation, eligible participants for the case series had used methylphenidate for over three years; were aged over six years when starting methylphenidate; had received at least one assessment of intellectual ability prior to starting methylphenidate and two assessments after; and were not lost to follow up or discharged from the paediatric neuro-oncology service. The decision to exclude children under six was made to reduce measurement bias by allowing for consistency of the assessment battery with that used at the Year 1 follow-up. To control for selection bias, all participants who met the eligibility criteria were invited via letter to consent to inclusion. Six patients were included in the final case series, including all patients with over three years of use of methylphenidate.

### 2.3. Measures

Measures of general intellectual ability were provided using the Wechsler Intelligence Scales for Children (WISC-V^UK^). Follow-up assessments were provided using the WISC-V^UK^ or the Wechsler Adult Intelligence Scales (WAIS IV^UK^) if participants were aged over 16 years and 11 months. The WISC-V^UK^ consists of 10 subtests that yield five indices: Verbal Comprehension, Visual Spatial, Fluid Reasoning, Working Memory, and Processing Speed. Seven core subtests are combined to produce a Full-Scale IQ (FSIQ). Intelligence tests were administered at diagnosis, at the methylphenidate treatment baseline, at 12 months (±3 months) follow-up, and yearly thereafter. The current case series describes the assessment of intelligence provided at six years old or as soon after six years as the child was diagnosed (D1), baseline immediately prior to use of methylphenidate (B1), at one year on methylphenidate (T1), and three years on methylphenidate (T2). Measures of attention and HRQoL collected from this cohort are described in detail in previous studies [7,8,14], using the Test of Everyday Attention for Children (TEACh-2) [28], the SNAP IV [29], and the PEDS-QL [30]. Side effects were measured using Barkley’s Stimulant Side Effect Rating Scale [31] and via clinical interview. Side-effect data were gathered at baseline, at 6 weeks (±2 weeks), at 6 months (±2 weeks), at 12 months (±3 months), and at 36 months (±6 months). Qualitative data were collated from patients’ historical clinical notes.

### 2.4. Procedures

The methylphenidate dose was determined following the British National Formulary—Child (BNFC) guidelines, within the range indicated by the RCT of Conklin et al. [5,32]. This resulted in a starting dose of 2.5 mg immediate-release methylphenidate hydrochloride twice a day for children 15–20 kg, 5 mg twice a day for those 21–30 kg, and 10 mg twice a day for those above 30 kg. The optimal dose was determined via the positive movement of the child’s attentional scores toward the level of their premorbid intellectual ability. Once the appropriate level of immediate-release methylphenidate was identified, patients were converted to an equivalent modified-release preparation as per the BNFC. All participants had been transferred to long-acting methylphenidate for a minimum of 18 months at the time of inclusion. Ongoing assessment and observation of height, weight, heart rate, and blood pressure were conducted following the NICE Guidelines [15].

### 2.5. Analyses

Linear mixed-effects model analyses (LMMs) were conducted to assess group changes in intellectual performance over the four assessment points (D1, B1, T1, and T2). LMMs offer several important and relevant strengths: they are well suited for analysing data collected over repeated assessments, the use of random effects can account for individual variability, and model flexibility enables valuable between-group analyses (i.e., between time points). To accommodate any treatment-related variability in intellectual trajectory, LMM analyses were conducted both with and without D1 scores. Each LMM was run in R (Version 4.3.0) using the ‘lme4′ package [33]. Significance was calculated using the ‘lmerTest’ package, which estimates degrees of freedom and *p*-values based on Satterthwaite’s method [34]. Secondary LMM analyses were performed with time treated as a categorical predictor to identify any specific assessment points that differed significantly from D1 or B1.

The Reliability Change Index (RCI) [35] (standardised difference score) was used to calculate the response of individual patients to methylphenidate using the formula RC=(X2−X1)/Sdiff, where *Sdiff* is calculated from the Standard Error of Measurement: *Sdiff* = √2(SE)2. The use of RCI analyses allowed for the identification of any change greater than the level already expected in a child maturing over a set period. Reliability data were gained using the ‘Clinical Sample’ standardisation group from the WISC V technical manual.

### 2.6. Ethical Approval

Ethical approval for this case series was sought from the Newcastle Upon Tyne Hospitals NHS Foundation Trust research team. Written informed consent was provided from all participants over 18 years old at the time of analyses. Written informed consent was sought from parents/guardians of all participants under 18 years, with assent sought from their child/charge.

## 3. Results

Six patients were eligible for inclusion in the case series according to the length of time they had received methylphenidate (see Table 1). The families of all eligible patients gave consent for inclusion. All had commenced methylphenidate treatment between April 2017 and November 2019. All patients showed improvement on measures of attention following the administration of methylphenidate (see [14] for full data on attentional gains). The mean age of participants in this case series was 14.7 years (range 12.5–18.4). The mean age at diagnosis was 4.5 years (range 1–12). The mean age at the start of methylphenidate treatment was 9.9 years (range 7.1–14.3). The mean length of time on methylphenidate was 4.8 years (range 3.3–5.6). The mean length of time between the tumour diagnosis and the start of methylphenidate was 5.4 years (range 2.3–8.2). Patients were in receipt of prescriptions for sustained-release methylphenidate ranging from 27 mg to 54 mg.

Patient A was diagnosed with a left thalamic WHO Grade 2 low-grade glioma with associated hydrocephalus at the age of 2 years. This was treated surgically, resulting in right-sided hemiplegia and hemianopia. Progression of the residuum was treated with further surgery, chemotherapy, and cranial radiotherapy to 50.4 Gy. The patient commenced methylphenidate at 9.8 years, continuing this to the time of analysis at 15.4 years (5.6 years on methylphenidate). While initial measures of attention showed only a mild impact upon attention, the assessment showed Patient A to have significant slowing in processing speed function, and thus methylphenidate was trialed. Reliable Change Index analyses found no benefit of methylphenidate on working memory, processing speed, or intellectual function.

Patient B was diagnosed with a left occipital WHO Grade 3 ependymoma at the age of 5 years. This was treated with surgery and with radical radiotherapy to the left occipital region to 54 Gy. Recurrence was treated with further radical radiotherapy to the left occipital region to 54 Gy. This patient commenced methylphenidate at 10.4 years and continued this at the time of analysis when he was 15.8 years (5.4 years on methylphenidate). Following an uneventful start to the use of methylphenidate, Patient B later reported a significant loss of appetite at lunchtime when using methylphenidate. This was managed while on short-acting methylphenidate by increasing his food intake in the evening. This method was ineffective once he moved on to the sustained-release preparation, during which he did not feel hungry at any time after breakfast. Patient B elected to stop taking methylphenidate at the weekend or during the school holidays. Despite this severe side effect, at no point did the overall SSERS score show an increase in the overall level of side effects. Reliable Change Index analyses showed improvement in working memory and intellectual function.

Patient C was diagnosed with a posterior fossa Grade 3 ependymoma with an associated hydrocephalus at the age of 1 year. This was treated surgically, involving resection of the tumour, a ventriculostomy, and proton beam radiotherapy to 54 Gy. Surgery resulted in the presentation of a mild posterior fossa syndrome. Patient C was then treated with chemotherapy. Following the measurement of a significantly decreased processing speed and lower level of attentional ability than would be predicted in a boy of his intellectual ability, Patient C started methylphenidate at 9.2 years (6.8 years after completion of chemotherapy) and continued this at the time of analysis when 12.5 years old. Reliable Change Index analyses showed improvement in working memory and intellectual function.

Patient D was diagnosed with a midline cerebellar Grade 4 medulloblastoma and associated hydrocephalus at the age of 12 years. This was treated surgically, involving the resection of the tumour and insertion of a ventricular shunt. He was treated further with craniospinal radiotherapy to 36 Gy with a tumour bed boost of 19.8 Gy and chemotherapy. Patient D also has Type I diabetes. He started methylphenidate at 14.3 years and continued this at the time of analysis when he was 18.4 years (4.1 years). Patient D reports perceiving the benefit of the methylphenidate on the ability to focus on schoolwork and is keen to continue using the medication for as long as possible via our clinic. Methylphenidate will be discontinued when he meets any of the following criteria: (a) parental or patient choice to stop, (b) unmanaged side-effects, or (c) patient finishes full-time education or has completed initial induction into apprenticeship/workplace. Reliable Change Index analyses showed improvement in the processing speed of methylphenidate but no change in working memory or intellectual function.

Patient E was diagnosed with a fourth ventricular Grade 2 ependymoma at the age of 2 years. This was treated with surgery and with the ‘Baby Brain’ protocol for chemotherapy treatment. Progression of the ependymoma was treated with proton beam radiotherapy. A new fourth ventricular tumour (Grade 3 ependymoma) was identified at the age of 4 years; this was treated with further proton beam radiotherapy and later with radical radiotherapy to the ventricular region to 48 Gy. Patient E started methylphenidate at 7.1 years and halted this at 12.5 years (5.4 years). The treatment was stopped as parents felt that they no longer observed intellectual or social benefit to Patient E, who, by this time, was predominantly schooled in the home. Reliable Change Index analyses showed improvement in working memory, processing speed, and intellectual function.

Patient F was diagnosed with a midline posterior fossa WHO Grade 4 anaplastic medulloblastoma with associated hydrocephalus at the age of 5 years. This was treated surgically, resulting in self-resolving ataxia and diplopia with spinal radiotherapy to 24 Gy, whole brain radiotherapy to 23.4 Gy, and posterior fossa boost to 30.6 Gy, and with chemotherapy. Patient F commenced methylphenidate at 8.6 years and continued this at the time of analysis when he was 13.5 years (4.9 years). Reliable Change Index analyses showed no change in working memory or processing speed and an improvement in intellectual function.

### 3.1. Group Performance

Assessment of intellectual ability was measured at four time points: following diagnosis as soon as 6 years of age (D1), baseline pre-methylphenidate (B1), at one year (T1), and at three years (T2). Scores gained are shown in Table 2. As anticipated, there were significant decreases in Full-Scale IQ (β = −7.33, SE = 2.93, *t* = −2.50, and *p* = 0.03) reported between diagnosis/early assessment (D1) and our baseline pre-methylphenidate (B1) assessment.

After starting methylphenidate, results show group-level raw score increases in verbal comprehension, fluid reasoning, working memory, processing speed, and full-scale IQ. Linear mixed-effects analyses of scores between B1 (baseline pre-methylphenidate) and T2 (at least three years using methylphenidate) did not show increases in mean group scores to be a statistically significant benefit. Consideration of individual performance using the Reliable Change Index showed an increase in working memory scores for three participants (participants B, C, and E), a benefit to processing speed for two participants (participants D and E), and a benefit to FSIQ for four participants (participants B, C, E, and F). The majority of participants who showed a benefit to FSIQ and processing speed demonstrated this via one-year post-methylphenidate (B1 to T1 comparison); however, the majority of those showing benefit to working memory did not show benefit as measured using RCI analysis until the three-year point (T2). Long-term increases in processing speed and working memory are consistent with responses found in some individuals with ADHD [36] and those with mild traumatic brain injury [37].

### 3.2. Side Effects

Side effects were assessed using Barkley’s Stimulant Side Effect Rating Scale. One participant reported increasing significantly lowered appetite (Patient B). This patient was offered the choice to halt methylphenidate, choosing instead to pause methylphenidate at weekends and holidays to support normal weight gain. Despite the significant decrease in appetite, Patient B’s SSERS side effect rating score appeared unaffected by the use of methylphenidate due to his improvement on non-appetite items. No other unwanted side effects were identified in any patient in this case series.

## 4. Discussion

Methylphenidate is an increasingly viable candidate in the management of attentional deficit in paediatric brain tumour survivors; however, little is known about its medium to long-term utility. Theoretical models such as that of Palmer suggest that management of attentional deficit may yield longer-term benefits to downstream intellectual and academic functions [11]. Studies within the ADHD population, however, suggest that methylphenidate lacks evidence to support these functions over the long term at best and potentially causes under-recognised side effects at worst. The current case series describes the trajectory of intellectual development in six survivors of a paediatric brain tumour over a minimum of three years of use of methylphenidate. These patient data, alongside a discussion of learning points from our previous studies, were used as a conduit for the identification of questions relating to the use of methylphenidate in a paediatric brain tumour. While recognising the methodological limitations inherent in the case series model, we used this case series to identify key questions to interrogate future data on long-term outcomes.

Our clinical case series highlights a number of unanswered questions with respect to the use of methylphenidate in the paediatric brain tumour population. (1) Is medium to long-term use of methylphenidate associated with later benefits to intellectual function in this population? While mean scores indicate a relatively consistent trend towards improved or maintained intellectual performance across the assessment period, linear mixed-model analysis did not identify a statistically significant benefit of methylphenidate on intellectual ability. Reliable Change Index analyses found an improvement in the intellectual function of four of the six patients in this case series. It is possible that methylphenidate is associated with the preservation of rather than an increase in long-term intellectual function. By mitigating the level of impairment to processing speed and attention, methylphenidate may act to preserve the developmental trajectory of downstream functions that would otherwise decline. Our future fully powered study using clinical control group data will consider the trajectories of decline and plateau between methylphenidate and non-methylphenidate groups;

(2) Which patients are the best candidates for use of methylphenidate? Based on Conklin et al., children with a severe attentional deficit find greater benefit of methylphenidate than children whose deficits are mild [6]. Patient E provides a helpful example of this, showing significantly lowered attention and processing speed prior to the use of methylphenidate and good clinical gains, as shown using Reliable Change Index analyses. It must be noted, however, that a significant benefit of methylphenidate can be found in patients whose attentional function is only mildly impaired. Kahalley et al. found a significant proportion of paediatric brain tumour survivors to have under-recognised attentional difficulties, as these presented differently to children with ADHD [38]. Further exploration with a larger clinical sample would allow for the analysis of the factors associated with the benefit of methylphenidate;

(3) What are the most effective timings for treatment initiation and discontinuation in this population? Whilst there is strong evidence of benefit to attention and the processing speed of methylphenidate in the initial months of use, studies in ADHD show a reduction in the efficacy of treatment over time and a lack of evidence for the benefit to academic attainment or intellectual development. Optimal timings for initiation and discontinuation must be identified, and the relationship between relevant factors (i.e., age at injury) with these timings. Based on the ADHD population, it is possible that children who are younger at the commencement of methylphenidate will show greater benefit from a longer period of use compared to older children [22]. With increasing experience in the use of methylphenidate in our own clinic, we are treating patients as soon as the deficit is identified and thus now treat patients at an early stage. Patient C suggests the potential utility of finding benefit to starting methylphenidate even some years after treatment (6.8 years post-treatment). At the other end of the treatment journey, Patient D illustrates some of the questions raised in the discontinuation of methylphenidate in this population. Frequently our older patients (and family) wish to continue the treatment that they value; however, as clinicians, we are limited by the lack of evidence base for the continued use of methylphenidate in this population and lack an established route for long-term prescribing for former paediatric patients who are discharged from oncology. Future research comparing treatment initiation and discontinuation times and optimal treatment duration of younger versus older survivors of a paediatric brain tumour would be of clinical utility;

(4) How early is methylphenidate helpful in the treatment pathway? It is possible that there may be a role for methylphenidate in the prophylaxis of attentional late effects. As demonstrated in intellectual scores, all patients showed an initial decline in performance in a number of domains following cancer treatment. Our hypothesis would be that the earliest possible identification and treatment of attentional deficit would be maximally protective against a future overall decline in intellectual development. We are also curious about the potential utility of methylphenidate given the pre-symptomatically in this population. Given that identification of attentional difficulties occurs following the observed functional consequences of an attentional deficit (e.g., declining academic attainment and instances of ‘memory’ failure), it is possible that the pre-symptomatic use of methylphenidate in a carefully targeted subset of survivors might offer benefit. Pre-clinical studies will be required to define the optimal timing and efficacy of prophylactic methylphenidate administration;

(5) Are we failing to effectively measure treatment-related side effects? The emergent literature on the long-term effect of methylphenidate in ADHD suggests a tendency for studies to overestimate the potential benefit of methylphenidate and to under-value the high level of ‘non-serious’ side effects [26]. Our study of the initial 12 months of methylphenidate treatment was consistent with that of Conklin et al. [4], finding a minimal side-effect profile [14]. It is possible, however, that side effects are experienced but not identified via the side-effect measure used. Studies of methylphenidate frequently use the Stimulant Side Effect Rating Scale (SSERS) to assess adverse effects [31]. Using the SSERS, some children with ADHD showed a reduction in ‘stimulant side effects’ once using stimulant medication [39]. Several SSERS items rating emotional symptoms are often improved via methylphenidate [40,41]. This phenomenon may mean that the SSERS underestimates adverse effects [42]. This is seen in the case of Patient B, whose reduced appetite was masked by improvement on other items. This suggests that the SSERS is a sub-optimal measure and that future development of an effective side-effect measure is required;

(6) Could methylphenidate be used as part of a rehabilitative pathway? While the current case series addressed solely methylphenidate, research addressing the potential utility of methylphenidate when used in a treatment pathway, including neuroprotective and/or anti-senescence drugs, would be of interest. Looking outside of pharmaceutical interventions, further research might identify helpful neuropsychological rehabilitative practices to be used as an adjunct to methylphenidate in managing cognitive late effects.

### Strengths and Limitations

Our case series is subject to a number of methodological limitations, most notably the restricted sample size, which significantly limits the applicability of our findings. While the case series design offers scope for hypothesis generation, benefits are clearly countered by the lack of a comparison group, limited capacity for generalisation, and an inability to claim causality [43]. Our demographic information was limited, meaning that factors with potential relevance to clinical outcomes, such as the socio-economic, ethnic, or cultural status of a family, were not described [44,45]. Further, the commercial reliability data used to calculate RCI thresholds do not specify a test–retest timescale; thus, this data may be suboptimal in informing our RCI calculations [46]. Our study does, however, provide a preliminary description of data pertaining to the longer-term use of methylphenidate and is the first to address the utility of methylphenidate in neuro-oncology patients over 12 months of use. We have identified six topics of importance, laying the groundwork for further studies in the survivorship community.

## 5. Conclusions

This clinical case series describes the medium-term use of methylphenidate by six survivors of a paediatric brain tumour and provides a conduit for the identification of questions for further investigation. Some of these questions may be answered reasonably easily. Our team is currently engaged in a longitudinal prospective study using a clinical and synthetic control. Based on questions raised by our clinical case series, we will examine whether methylphenidate reduces the extent of treatment-related deficits in intellectual function. We will also explore the use of alternative measures of side effects in addition to our planned use of the SSERS. Other questions pose greater challenges. Identifying optimal timing for methylphenidate discontinuation requires studies that negotiate the ethics of withholding effective treatment. Other questions, such as the use of methylphenidate as a prophylactic or as part of a treatment pathway alongside other pharmacological and non-pharmacological interventions, still require costly pre-clinical studies. We are optimistic that continued exploration and collaboration will allow us to fully describe an effective interventional pathway for managing long-term late effects in brain tumour survivorship in which methylphenidate may play a role.

## Figures and Tables

**Table 1:** Demographic and clinical characteristics of the sample.

Patient	Sex	Age at Diagnosis (Years)	Ethnicity	Diagnosis Including Comorbidities	Treatment	Age at MPH Start	Age Now	Time Using MPH *
A	F	2	White British	Left thalamic Grade 2 Low-Grade Glioma (recurred), Hydrocephalus, Right-sided Hemiplegia, and Hemianopia	Surgery Ventriculostomy Vincristine/Carboplatin Bevacuzimab/Irinotecan RT 50.4 Gy	9.8	15.4	5.6
B	M	5	White British	Left occipital Grade 3 Ependymoma	Surgery ×2 Focal RT 54 Gy Re-focal RT 54 Gy	10.4	15.8	5.4
C	M	1	White British	Posterior Fossa Grade 3 Ependymoma Hydrocephalus Mild PFS	Surgery Ventriculostomy PBT 54 Gy Cisplatin/Methotrexate Cyclophosphamide Carboplatin/Vincristine	9.2	12.5	3.3
D	M	12	White British	Midline cerebellar Grade 4 Medulloblastoma Hydrocephalus Type I Diabetes	Surgery Ventricular shunt RT 36 Gy Tumour bed boost 19.8 Gy Cyclophosphamide/Cisplatin Vincristine	14.3	18.4	4.1
E	F	2	White British	4th ventricular Grade 2 Ependymoma (recurred). Emergence of Grade 3 Ependymoma	Surgery Cisplatin/Methotrexate Cyclophosphamide Carboplatin/Vincristine PBT, re-PBT, Focal RT 48 Gy	7.1	14.3 **	5.4
F	M	5	White British	Midline Posterior Fossa Grade 4 Medulloblastoma Hydrocephalus	Surgery Cyclophosphamide/Cisplatin Vincristine Spine RT 24 Gy Whole brain RT 23.4 Gy Tumour bed boost 30.6 Gy	8.6	13.5	4.9

Note. MPH = methylphenidate, RT = conventional radiotherapy, PBT = proton beam radiotherapy, PFS = posterior fossa syndrome/cerebellar mutism, Gy = gray * For patients still in receipt of a prescription of methylphenidate, ‘length of time’ was calculated to the time of analysis (August 2023). ** Patient E discontinued methylphenidate after 5.4 years.

**Table 2:** Group mean index scores of intellectual ability and cognitive processing skills at D1, B1, T1, and T2 and group comparisons.

	At Diagnosis (D1)	Pre-MPH (B1)	One Year MPH (T1)	Three Years MPH (T2)	B1 vs. T1	B1 vs. T2	Total ^b^
Intellectual Index	Mean (SD)				*p* Value		
Verbal Comprehension	97.2 (7.5)	89.5 (9.1)	92.5 (8.2)	92.4 (6.2)	0.35	0.27	0.23
Visual–Spatial	99.3 (8.4)	100.1 (8.3)	95.5 (4.8)	96.2 (8.4)	0.25	0.34	0.31
Fluid Reasoning	97 (6.1)	91.3 (10.2)	96.5 (11)	93.4 (10)	0.31	0.56	0.51
Working Memory	93.5 (10)	93.2 (8.8)	94.5 (12.6)	98.5 (13.2)	-	-	0.75 ^a^
Processing Speed	93.5 (4.7)	83.8 (7.4)	84.5 (10.1)	89.5 (11.4)	0.91	0.37	0.35
Full-Scale IQ	94.8 (4.9)	87.5 (8.6)	90.5 (8.3)	92.2 (6)	0.32	0.20	0.17

^a^ During statistical analysis, working memory data failed to align appropriately with a random effects model, in turn leading to a singularity issue. The LMM was simplified by removing random effects and fitting a fixed-effect model. The associated overall *p*-value for working memory, therefore, uses a fixed-effect model. ^b^ Total represents the significance value obtained from the overall random-effects model without the inclusion of D1 or time treated as a categorical predictor. Notes: SD = standard deviation. IQ = intelligence quotient. MPH = methylphenidate. All scores gained via the WISC IV or WISC V at diagnosis. All scores gained via WISC V at B1 and T1. All scores at T2 gained via WISC V or WAIS IV. WISC IV, V, and WAIS IV: mean = 100, SD = 15. Scores of 80–90: low average 90–110: average.

## Data Availability

The data presented in this study are available on request from the corresponding author. The data are not publicly available due to patient confidentiality.

## Appendix A

**Table A1:** Eligibility criteria for survivors of a paediatric brain tumour for methylphenidate.

Inclusion Participant has been treated for CNS tumour in the previous 10 years, counting from diagnosis date; Patient is aged between 6 years 0 months and 14 years 0 months at the start of the trial; Has been off therapy/active treatment for CNS tumour for 12 months at the start of the trial and has a likely life expectancy of >5 years; No known signs of clinical or radiological tumour progression; English is the sole or primary language (enables the provision of valid psychometric assessment); Patient and family have provided assent/consent for inclusion in the trial; Neurocognitive impairment of processing speed or attention as assessed by: (a) Clinically significant loss of previous function in processing speed and/or attention as shown by repeat psychometric assessment or estimate of premorbid ability. Clinical significance will be determined by the Site Lead; OR (b) Discrepancy between the General Ability Index (GAI) and Cognitive Processing Index (CPI) (GAI > CPI) that is statistically significant at *p* = 0.05 for age (see WISC V Administration and Scoring Manual pp. 320 for critical values tables), or GAI and processing speed (GAI > PSI) of ten index score points or more.
Exclusion (i) Contraindications to methylphenidate medication as per current BNF for Children (BNFC) and NICE Guideline 87: (a) Family history of tic disorder; (b) Glaucoma (history in family from an early age); (c) Current antidepressant or anxiolytic use; (d) Significant problems maintaining weight or previous eating disorder; (e) History of substance misuse; (f) History of recent poorly controlled seizures; (g) History of cardiac issues; (h) Pregnancy: while pregnancy and breastfeeding are not listed as contraindications in the BNFC, methylphenidate is listed as ‘Limited experience—avoid unless the potential benefit outweighs risk’. Prospective participants known to be pregnant or breastfeeding at screening/registration will not be enrolled in the trial. (i) Significant mental health difficulties (e.g., clinically severe depression, psychosis); (ii) Diagnosis of Attention Deficit/Hyperactivity Disorder or Autism Spectrum Disorder; (iii) Full Scale Intelligence Quotient (FSIQ) of <50; (iv) Concerns about family ability to safely store or administer methylphenidate or to report side effects appropriately/Concerns about familial substance misuse.
